# Protocol for a randomised controlled trial to investigate the effects of vitamin K2 on recovery from muscle-damaging resistance exercise in young and older adults—the TAKEOVER study

**DOI:** 10.1186/s13063-022-06937-y

**Published:** 2022-12-20

**Authors:** Hannah Lithgow, Lynsey Johnston, Frederick K. Ho, Carlos Celis-Morales, James Cobley, Truls Raastad, Angus M. Hunter, Jennifer S. Lees, Patrick B. Mark, Terry J. Quinn, Stuart R. Gray

**Affiliations:** 1 School of Cardiovascular and Metabolic Health, University of Glasgow, Glasgow, G12 8TA UK; 2 School of Life Sciences, University of Glasgow, Glasgow, UK; 3 School of Health and Wellbeing, University of Glasgow, Glasgow, UK; 4 Division of Biomedical Sciences, University of Highlands and Islands, Inverness, UK; 5 Department of Physical Performance, Norwegian School of Sports Science, Oslo, Norway; 6 Department of Sprots Science, Nottingham Trent University, Nottingham, UK; 7 Department of Health Promotion and Rehabilitation, Lithuanian Sports University, Kaunas, Lithuania

**Keywords:** Resistance exercise, Muscle damage, Recovery, Inflammation, Vitamin K

## Abstract

**Background:**

Regular participation in resistance exercise is known to have broad-ranging health benefits and for this reason is prominent in the current physical activity guidelines. Recovery after such exercise is important for several populations across the age range and nutritional strategies to enhance recovery and modulate post-exercise physiological processes are widely studied, yet effective strategies remain elusive. Vitamin K2 supplementation has emerged as a potential candidate, and the aim of the current study, therefore, is to test the hypothesis that vitamin K2 supplementation can accelerate recovery, via modulation of the underlying physiological processes, following a bout of resistance exercise in young and older adults.

**Methods:**

The current study is a two-arm randomised controlled trial which will be conducted in 80 (40 young (≤40 years) and 40 older (≥65 years)) adults to compare post-exercise recovery in those supplemented with vitamin K2 or placebo for a 12-week period. The primary outcome is muscle strength with secondary outcomes including pain-free range of motion, functional abilities, surface electromyography (sEMG) and markers of inflammation and oxidative stress.

**Discussion:**

Ethical approval has been granted by the College of Medical Veterinary and Life Sciences Ethical Committee at the University of Glasgow (Project No 200190189) and recruitment is ongoing. Study findings will be disseminated through a presentation at scientific conferences and in scientific journals.

**Trial registration:**

ClinicialTrials.gov NCT04676958. Prospectively registered on 21 December 2020.

## Administrative information

Note: the numbers in curly brackets in this protocol refer to SPIRIT checklist item numbers. The order of the items has been modified to group similar items (see http://www.equator-network.org/reporting-guidelines/spirit-2013-statement-defining-standard-protocol-items-for-clinical-trials/).

**Table:** 

Title {1}	Protocol for a randomised controlled trial to investigate the effects of vitamin K2 on recovery from muscle-damaging resistance exercise in young and older adults—the TAKEOVER study
Trial registration {2a and 2b}.	Prospectively registered on 21^st^ December 2020, trial registration clinicialtrials.gov ID NCT04676958 https://clinicaltrials.gov/ct2/show/NCT04676958
Protocol version {3}	Version 1. 3^rd^ June 2020
Funding {4}	The study is funded by a grant from Kappa Biosciences to University of Glasgow.
Author details {5a}	Hannah Lithgow^1^, Lynsey Johnston^1^, Fred Ho^2^, Carlos Celis-Morales^1^, James Cobley^3^, Angus Hunter^4^, Truls Raastad ^5^, Jennifer Lees^1^, Patrick Mark^1^, Terry Quinn^1^, Stuart R Gray^1,6^ ^1^ School of Cardiovascular and Metabolic Health, University of Glasgow ^2^ School of Health and Wellbeing, University of Glasgow ^3^ Division of Biomedical Sciences, University of Highlands and Islands ^4^ Department of Sprots Science, Nottingham Trent University ^5^ Department of Physical Performance, Norwegian School of Sports Sciences ^6^ Department of Health Promotion and Rehabilitation, Lithuanian Sports University HL, TQ, and SRG were responsible for the conception of the work. All authors contributed to the study design. FH and CCM were responsible for the statistical analysis plan for the study. SRG drafted the manuscript and all other authors revised it critically for important intellectual content and give final approval of the version to be published and agree to be accountable for all aspects of the work in ensuring that questions related to the accuracy or integrity of any part of the work are appropriately investigated and resolved.
Name and contact information for the trial sponsor {5b}	Dr Debra Stuart debra.stuart@glasgow.ac.uk College of Medical, Veterinary & Life Sciences Room 327 Wolfson Medical School Building University Avenue GLASGOW G12 8QQ +44 (0)141 330 4539
Role of sponsor {5c}	The sponsor had no role in the study design; collection, management, analysis, and interpretation of data; writing of the report; and the decision to submit the report for publication, and will have no ultimate authority over any of these activities.

## Introduction

### Background and rationale {6a}

The main role of the muscle is to allow body movements via the generation of force, with the importance of this highlighted in conditions associated with muscle mass loss, such as sarcopenia [1]. Additionally, skeletal muscle has a critical, but often overlooked, role in metabolism [2]. For example, skeletal muscle is the primary protein store in the body and, during starvation [3] or conditions such as AIDS, can provide gluconeogenic precursors which are crucial for survival [4]. On top of this, as muscle is the primary site for glucose disposal in the body, it is therefore important in metabolic conditions such as diabetes [5]. Extending this further, the importance of muscle in lifelong health is reflected by data demonstrating the association of muscle mass/function with mortality [6]. Taking this evidence together, this indicates that the maintenance of muscular strength is of clear importance for public health.

The most efficacious way to increase muscle mass is via the performance of resistance exercise which has broad-ranging health benefits [7], and it is therefore not surprising that such exercise appears in the current physical activity recommendations [8]. Very few people, however, meet these muscle-strengthening guidelines with participation rates much lower (~30%) than for the aerobic (~65%) recommendations [9]. Whilst there are very few side effects of such exercise, one of the acute effects seen primarily in people unaccustomed to such exercise, or who have not carried it out for a whilst, is muscle damage. This will result in a decrease in muscle function, swelling and an increase in circulating muscle proteins (such as creatine kinase (CK)), inflammatory cytokines (such as interleukin-6 (IL-6)) and markers of oxidative stress [10]. This can affect subsequent participation in exercise due to residual loss of muscle function. This is relevant for many groups, for example, athletes in training and competition, recreational gym-goers’ ability to maintain attendance and older adults’ abilities to carry out everyday tasks/activities of daily living. With this in mind, there are many areas of research which aim to reduce these physiological responses to acute exercise with nutrition at the forefront of this endeavour [11].

Vitamin K includes a group of structurally related compounds named phylloquinone (vitamin K1) and menaquinones (vitamin K2s). Vitamin K was discovered by the Danish biochemist Henrik Dam in the 1930s, and he demonstrated that the product was involved in coagulation. Dam received the Nobel Prize in 1943 for his work on vitamin K. The vitamin K2s or menaquinones (MK-[n]) were discovered in the 1960s, of which MK-4 and MK-7 are the most studied. Vitamin K1 is regarded as the major dietary source of vitamin K, found in green vegetables such as broccoli, cabbage and spinach. Vitamin K2 is found in low quantities in cheese, egg yolk, meat and curd. The richest natural food source of vitamin K2 is the traditional Japanese natto made by fermented soybeans, providing a good source of MK-7. Natto is a popular food in Japan but is not highly appreciated elsewhere due to the taste. The MK-4 is registered as a drug in Japan for the treatment of osteoporosis. In Europe, two forms of vitamin K exist today in food supplements: vitamin K1 and vitamin K2 as MK-7.

One nutritional supplement which may be effective but which has very little research is vitamin K2. Although there has been little exercise and vitamin K2 work, there is evidence that vitamin K2 has important roles in redox balance and also in mediating inflammation. Vitamin K is an essential cofactor for gamma carboxylation of glutamic acid residues in many vitamin K-dependent proteins (VKDPs), required for the effective function of a range of proteins [12] including those involved in bone remodelling, vascular calcification, glucose handling, inflammation and neuromuscular function [13–19]. Low levels of dietary vitamin K intake are very common and associated with a higher risk of cardiovascular disease and osteoporosis [20, 21]. Due to these roles in redox balance and inflammation, processes linked to post-exercise muscle damage and recovery [10, 22–24], it is possible that vitamin K2 may play an important role in modulating the acute physiological responses to exercise.

The main aim of the current study, therefore, is to test the hypothesis that vitamin K2 supplementation can accelerate recovery, via modulation of the underlying physiological processes, following a bout of resistance exercise in young and older adults.

### Objectives {7}

Our primary aim is to test the hypothesis that vitamin K2 supplementation can accelerate recovery, via modulation of the underlying physiological processes, following a bout of resistance exercise in young and older adults.

### Trial design {8}

The current study is a two-arm randomised controlled trial comparing placebo with vitamin K2 supplementation. Eligible participants will be identified from local advertisements. Baseline data will be measured prior to randomisation, and follow-up data will be collected 12 weeks after randomisation adjusted for any delays in the start of the intervention post-randomisation.

### Patient and public involvement

There was no patient and public input in the design of the study protocol.

## Methods: participants, interventions and outcomes

### Study setting {9}

This is a single-centre, University of Glasgow, two-arm randomised controlled trial.

### Eligibility criteria {10}

Potential participants will receive a mailed prestudy invitation which will provide details on study information and details around eligibility. If still interested, potential participants will be invited to the research site where eligibility will be determined based on the inclusion and exclusion criteria (Table 1).

**Table 1:** Inclusion and exclusion criteria

In order to be considered eligible for participation in the study, they must:	
Criterion	Characteristics of eligible participants
1	Be male or female aged 18–40 years (young group) or ≥65 years (older group) at the time of consent
2	The participant is able and willing to sign the informed consent form
3	No plans to change lifestyle (activity and nutrition) during the study period
4	Not currently, or in the last year, participating in more than 1h per week of vigorous aerobic physical activity or any resistance exercise
5	BMI ≤ 30 kg/m^2^
6	Not have diabetes mellitus, severe cardiovascular disease, dementia, seizure disorders, liver disease, uncontrolled hypertension (>150/90mmHg at baseline measurement), cancer or cancer that has been in remission <5 years
7	Not have ambulatory impairments which would limit the ability to perform assessments of muscle function
8	Not be currently taking vitamin K2 supplements
9	Not be currently taking vitamin K antagonists/anticoagulants (e.g. warfarin)
10	Not be a current smoker
11	Not have a history of drug abuse
12	Not be currently taking medication known to affect muscle (e.g. steroids)

### Who will take informed consent? {26a}

Written consent will be obtained by a member of the study team during a visit to the research site for the baseline assessment, with no study procedures taking place until consent has been obtained. At the baseline visit, a member of the study team will confirm eligibility.

### Additional consent provisions for collection and use of participant data and biological specimens {26b}

Not applicable.

## Interventions

### Explanation for the choice of comparators {6b}

The comparator chosen is a placebo capsule without the active ingredient.

### Intervention description {11a}

#### Control

Participants in the control group will consume one 380-mg micro-crystalline cellulose tablet per day.

#### Vitamin K2

Participants in the vitamin K2 will consume a 380-mg micro-crystalline cellulose tablet containing 240 μg vitamin K2 (MK-7) (K2VITAL® 0.2% DELTA powder) per day.

### Criteria for discontinuing or modifying allocated interventions {11b}

In accordance with the Declaration of Helsinki, participants can withdraw from the trial at any time for any reason. Furthermore, the investigator can withdraw a participant from the trial at any time if a withdrawal is considered in their best interest. Participants who have stopped the intervention prior to completion will be asked to follow study visits according to the protocol. Participants who choose to withdraw from the trial will be asked the reason why. However, it is emphasised that they are not obliged to state the reason.

### Strategies to improve adherence to interventions {11c}

Participants will be sent a text message every 2 weeks to check how they are getting on taking their supplements and to serve as a reminder to do so.

### Relevant concomitant care permitted or prohibited during the trial {11d}

Participants will be asked to maintain their usual physical activity and nutritional habits throughout the trial. Usual healthcare will also be maintained.

### Provisions for post-trial care {30}

There are no specific provisions for post-trial care within this study, other than access to usual healthcare.

### Outcomes {12}

Baseline characteristics will be collected—age, sex, blood pressure, weight, height, BMI, habitual diet, habitual physical activity levels, current medications and comorbidities.

All outcome measures will be assessed at baseline and 12 weeks in all participants. Where the post-exercise recovery of outcomes is detailed, these will be measured fasted at baseline (pre-exercise) after which an exercise bout will take place. The exercise will involve bilateral leg extension exercises at 70% of one-repetition maximum (1RM), with 5 sets of 15 repetitions performed. Each repetition will be performed for 4–2s in the concentric and 2s in the eccentric phase. If necessary, the concentric phase can be assisted. Following this, the same protocol will be performed for the bench press. A standardised snack will then be consumed. Further measurements will be made at 3h, 24h, 48h and 72h post-exercise.

#### Primary outcome measure

The primary outcome measure is the overall change in post-exercise recovery of knee extensor muscle maximal isometric torque from baseline to 12 weeks as shown in growth curve models.

#### Secondary outcome measures

Secondary outcome measures are the change post-exercise recovery from baseline to 12 weeks of:

- Pain-free range of motion
- Time to complete 5 chair rises
- Knee extensor surface electromyography (sEMG)
- Systemic markers of inflammation (e.g. interleukin-6 and interleukin-1β)
- Systemic markers of oxidative stress (e.g. peroxiredoxin 3 redox state and F2-isoprostanes)

And resting levels of:

- Vitamin K levels
- Carboxylated osteocalcin
- Uncarboxylated osteocalcin
- Carboxylate/uncarboxylated osteocalcin
- Carboxylated matrix gla-protein
- Dephosphorylated-uncarboxylated matrix gla-protein

#### Exploratory outcome measures

Exploratory outcome measures are the change from baseline to 12 weeks of:

- Lean and fat mass (bioelectrical impedance)
- Blood glucose
- Blood lipids
- Plasma insulin
- Vastus lateralis muscle thickness
- Substrate utilisation (carbohydrate and fat oxidation) during submaximal exercise

### Participant timeline {13}

**Fig. 1:**
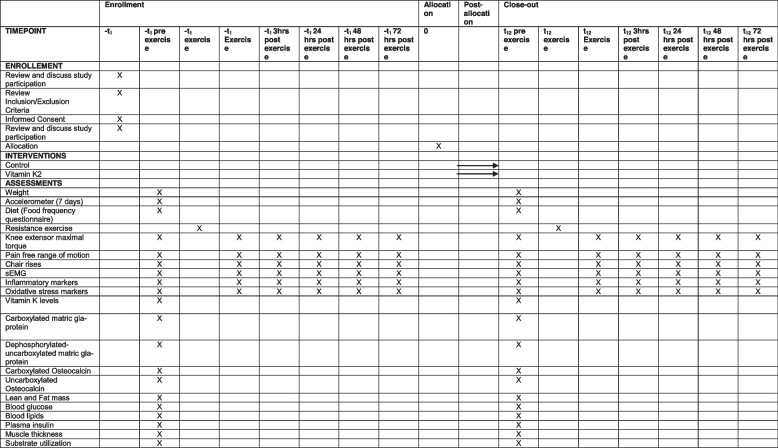
Schedule of enrolment, interventions and assessments

The schedule of enrolment, interventions and assessments is shown in Fig. 1.

### Sample size {14}

We have based our sample size on being able to (within age groups) detect a physiologically relevant difference in muscle strength of 10% with an SD of 10%, based on data from within our laboratory, with a power of 80% and alpha level of 0.05. This would require a sample size of 17 in each group and so we will aim to recruit 20 participants to each group (within age groups) to account for dropout.

### Recruitment {15}

Older and young people will be recruited using various strategies.

- Using advertisements via multiple media outlets that include local newspapers such as the *Glasgow Evening Times* and via social media such as Twitter and Facebook
- Flyers will also be distributed, and posters placed in local public places such as supermarkets, clubs, churches, bowling centres and community centres
- Via local organisations, such as University clubs and local older adult groups

## Assignment of interventions: allocation

### Sequence generation {16a}

Following the baseline assessment, patients will be randomised to the control or vitamin K2 groups (1:1 ratio) using a computer-generated (sealedenvelope.com) randomisation sequence with randomly permuted blocks. Randomisation will be stratified by age group (18–40; ≥65 years) and by sex. The randomisation sequence will be generated by an independent statistician.

### Concealment mechanism {16b}

Participants, research staff and trial statistician will be blind to allocation. The randomisation sequence will be generated by an independent statistician and the sequence passed to independent staff within the manufacturing facility who will label the supplement packets with a participant number. Staff and participants will be blinded to this sequence and all data will be analysed blind to allocation.

### Implementation {16c}

Patients will be enrolled by a member of the research team, and following randomisation, the supplement packet for that participant ID will be given to them.

## Assignment of interventions: blinding

### Who will be blinded {17a}

Participants, research staff and statistician will be blind to allocation.

### Procedure for unblinding if needed {17b}

Unblinding will take place upon request of the participant or the clinical research staff. The independent statistician holding the list will be contacted by the research team and will disclose the allocation to that participant. This will be logged in the study database.

## Data collection and management

### Plans for assessment and collection of outcomes {18a}

All outcome data will be collected according to developed standard operating procedures.

### Plans to promote participant retention and complete follow-up {18b}

No further strategies, than those described to maintain engagement with intervention, are planned.

### Data management {19}

All data will be kept in a secure storage area with access only to the study staff.

### Confidentiality {27}

Access to the collated participants’ data will be restricted to the principal investigator and appropriate research study staff as required. All laboratory samples, completed forms, reports and other records will be identified using a unique participant ID number to maintain participant confidentiality.

### Plans for collection, laboratory evaluation and storage of biological specimens for genetic or molecular analysis in this trial/future use {33}

Blood samples will be collected via venepuncture and analysed in our clinical biochemistry laboratories.

## Statistical methods

### Statistical methods for primary and secondary outcomes {20a}

The data analysis will be performed by the trial statistician (FH) who will be blinded to the group assignment. Participants’ characteristics will be described using mean and standard deviation (or median and interquartile range for skewed no-normal variables) for continuous variables and frequency tables for categorical variables. The primary analysis would be based on multilevel growth curve models including baseline and all post-exercise recovery period measurements. The time variable will be modelled as a penalised spline if a nonlinear association between time and outcome was found. The group × time interaction would be the primary variable of interest, indicating overall differences between the recovery process. Additionally, for each of the outcomes and time points, two-sample *t*-tests will be used to compare between-group differences in baseline and in follow-up assessments. Cohen’s *d* will be calculated to quantify effect sizes.

### Interim analyses {21b}

No interim analysis is planned in the current study.

### Methods for additional analyses (e.g. subgroup analyses) {20b}

Subgroup analysis will be conducted for sex and age group (younger vs. older) and interactions between these subgroups and treatment groups will be tested.

### Methods in analysis to handle protocol non-adherence and any statistical methods to handle missing data {20c}

Adherence to supplements will be determined by capsule count following the completion of the intervention period. Non-adherence will be handled through the use of intention-to-treat analysis in the primary analysis, and we will also add an additional instrumental variable analysis to examine effect sizes if the participant is fully compliant with supplementation [25]. Missing data will be handled by baseline observation carried forward in the primary analysis. Due to the randomised design, multivariable models will not be used to adjust for confounding, and therefore, missing covariates will not impact the primary analysis.

### Plans to give access to the full protocol, participant-level data and statistical code {31c}

The data sets analysed and the codes used for these analyses will be available from the corresponding author on reasonable request.

## Oversight and monitoring

### Composition of the coordinating centre and trial steering committee {5d}

#### Principal investigator

- Design and conduct of the trial
- Preparation of protocol and revision
- Preparation of study materials and case report forms
- Organise steering committee meetings
- Publication of study reports

#### Trial steering committee (principal investigator, study coordinator, independent academic clinician)

- Agreement of the final protocol
- All lead investigators will be members
- Recruitment of patients and liaising with principal investigators
- Reviewing the progress of the study and if needed agreeing to changes to the protocol

#### Trial Management Committee (principal investigator, study coordinator, researchers)

- Study planning
- Adverse event reporting
- Responsible for trial master file
- Budget control
- Data verification

### Composition of the data monitoring committee, its role and reporting structure {21a}

Not applicable.

### Adverse event reporting and harms {22}

There are unlikely to be major safety issues with our nutritional supplement interventions. However, if any safety concerns or incidents arise, we will follow our standard operating procedures for reporting adverse events in a non-Clinical Trial of an Investigational Medicinal Product (non-CTIMP) study.

### Frequency and plans for auditing trial conduct {23}

Formal audits will occur every 6 months.

### Plans for communicating important protocol amendments to relevant parties (e.g. trial participants, ethical committees) {25}

Any amendments to the protocol will be approved by the local ethics committee prior to implementation. All investigators and patients enrolled in the trial will be informed.

### Dissemination plans {31a}

The study findings will be disseminated primarily via conference presentations and scientific papers.

## Discussion

The current trial is a single-centre, two-arm randomised controlled trial. It has been designed to test the effect of vitamin K2 supplementation on recovery after a bout of resistance exercise. We will also investigate the effects on the inflammatory and oxidative processes after resistance exercise. Such information will be of importance to adults, young and old, who are interested in modulating these processes and the speed at which they recover after a single session of resistance exercise—a form of exercise recommended in the current physical activity recommendations.

### Trial status

The study began recruitment in May 2021 and will be ongoing until August 2022 (anticipated). Current protocol: version 1 (03.06.2020).

## Data Availability

Any data required to support the protocol can be supplied on request.
